# CDK1 drives SOX9-mediated chemotherapeutic resistance in gastric cancer

**DOI:** 10.1186/s13046-025-03523-3

**Published:** 2025-10-08

**Authors:** Marwah Al-Mathkour, Zheng Chen, Julio Poveda, Longlong Cao, Oliver G. McDonald, Dunfa Peng, Mohammed Soutto, Zhibin Chen, Heng Lu, Yan Guo, Shria Kumar, Alexander Zaika, Silvia Giordano, Shoumin Zhu, Wael El-Rifai

**Affiliations:** 1 Department of Surgery, Miller School of Medicine, University of Miami, 1600 NW 10th Ave, Room 4007, Miami, FL 33136-1015 USA; 2 Department of Pathology and Laboratory Medicine, Miller School of Medicine, University of Miami, Miami, FL USA; 3 Department of Gastric Surgery, Fujian Medical University Union Hospital, Fuzhou, China; 4 Department of Veterans Affairs, Miami Healthcare System, Miami, FL USA; 5 Department of Microbiology and Immunology, Miller School of Medicine, University of Miami, Miami, FL USA; 6 Department of Public Health, Miller School of Medicine, University of Miami, Miami, FL USA; 7 Department of Medicine, Miller School of Medicine, University of Miami, Miami, FL USA; 8 Department of Oncology, University of Torino and Candiolo Cancer Institute, Candiolo, Italy; 9 Sylvester Comprehensive Cancer Center, Miller School of Medicine, University of Miami, Miami, FL USA

**Keywords:** Gastric cancer, CDK1, SOX9, BCL-xL

## Abstract

**Background:**

Gastric carcinoma ranks as the fifth most common cause of cancer-related mortality globally. Chemoresistance remains a critical barrier to treatment efficacy, driving poor survival outcomes in gastric cancer patients. Cyclin-dependent kinase 1 (CDK1) is overexpressed in several malignancies. SOX9 transcription factor plays critical roles in gastric tumorigenesis and therapeutic resistance. This study identifies a CDK1-SOX9-BCL-xL signaling axis as an important mediator of chemoresistance in gastric cancer.

**Methods:**

Bioinformatics and computational approaches were used for analysis of human and mouse public and local data sets. Chromatin immunoprecipitation (ChIP), western blotting, quantitative PCR (qPCR), immunofluorescence, and immunohistochemistry assays were applied in the study. The study utilized a number of in vitro models including cell lines and patient-derived tumoroids. The in vivo models included patient-derived xenograft (PDX), the *Tff1* knockout, and *Cdk1* conditional knockout mouse models.

**Results:**

Our study identified concurrent overexpression of CDK1 and SOX9 in gastric cancer patients. Genetic knockdown and pharmacological inhibition of CDK1 suppressed SOX9 protein levels and transcriptional activity in vitro and in vivo. Mechanistically, CDK1 regulates SOX9 through a miR-145-dependent epigenetic axis: CDK1-mediated phosphorylation and activation of DNMT1 to drive methylation-dependent silencing of miR-145, thereby relieving miR-145’s repression of SOX9. Strikingly, both CDK1 and SOX9 were upregulated in cisplatin-resistant gastric cancer cell lines. We further identified BCL-xL as a direct transcriptional target of SOX9, functionally mediating cisplatin resistance. CDK1 inhibition using dinaciclib re-sensitized resistant models to cisplatin by disrupting the CDK1-SOX9-BCL-xL pathway, underscoring its central role in chemoresistance. In PDX models, combining dinaciclib with cisplatin synergistically reduced tumor volume, and extended survival compared to monotherapies, highlighting the therapeutic potential.

**Conclusion:**

This study elucidates the epigenetic and transcriptional mechanisms driving the CDK1-SOX9-BCL-xL axis in gastric cancer chemoresistance. Pharmacological inhibition of CDK1 effectively disrupts this axis, restoring cisplatin sensitivity and suppressing tumor growth in gastric cancer models. The observed synergy between dinaciclib and cisplatin underscores a promising therapeutic strategy to overcome chemoresistance in gastric cancer.

**Graphical abstract:**

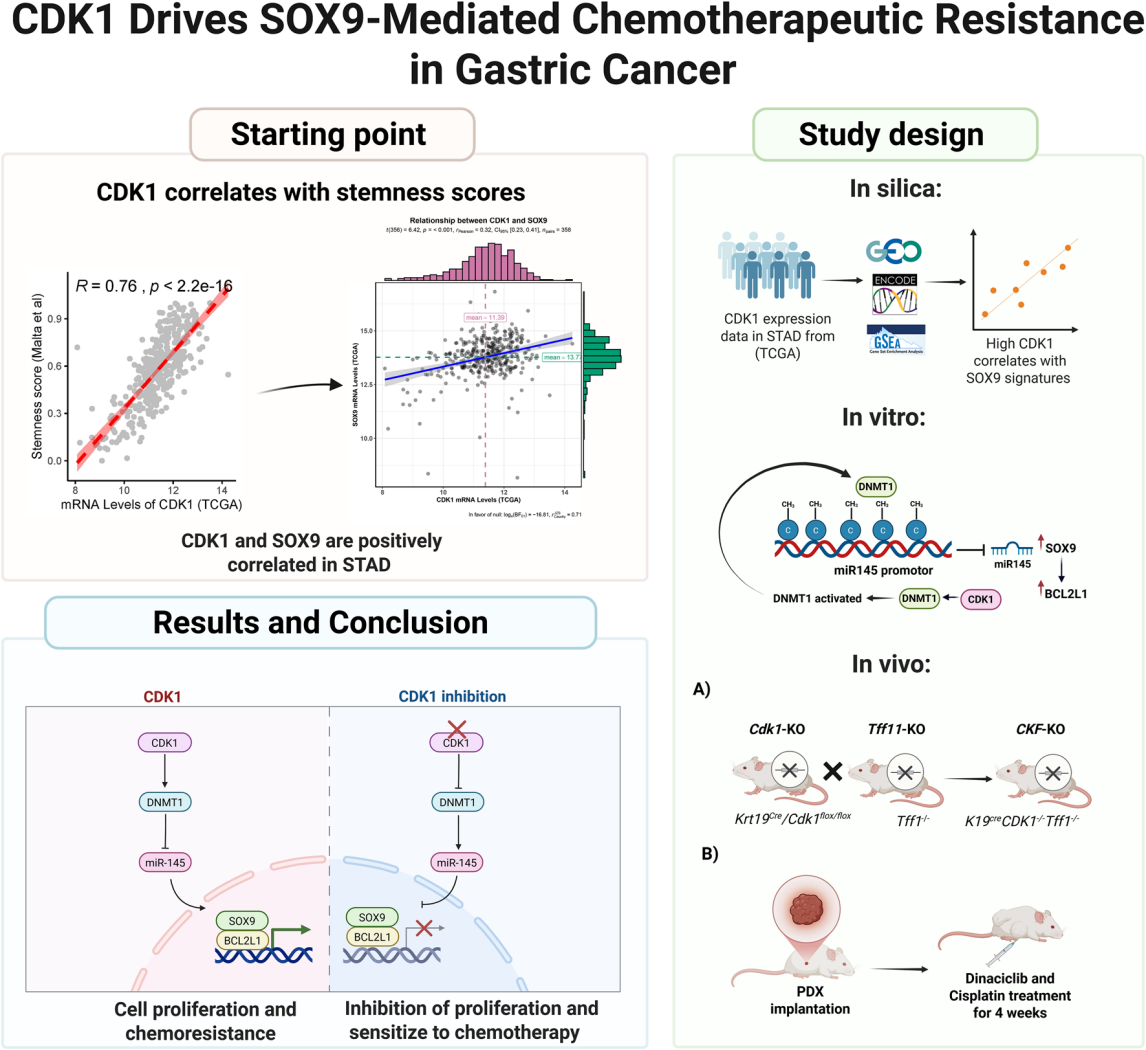

**Supplementary Information:**

The online version contains supplementary material available at 10.1186/s13046-025-03523-3.

## Background

Gastric cancer (GC) is the fifth leading cause of cancer-related deaths globally [1]. Despite advancements in chemotherapy regimens, a substantial proportion of GC patients either do not respond to treatment initially or develop resistance over time [2]. Understanding the mechanisms underlying chemoresistance is crucial for improving treatment efficacy and patient survival.

Cell cycle dysregulation, a hallmark of cancer, drives uncontrolled cell proliferation and tumor progression [3]. Among the regulatory proteins involved, cyclin-dependent kinase 1 (CDK1) is a key player in cell cycle control [4]. Emerging evidence indicates that CDK1 plays additional roles in cancer cells beyond cell cycle regulation, including the regulation of cell differentiation and stemness [5, 6]. In gastric cancer, CDK1 overexpression has been shown to activate β-catenin signaling [7]. Overexpression of CDK1 in cancer is associated with poor prognoses and has been linked to chemoresistance across various tumor types [8, 9]. Although, CDK1 overexpression is associated with poor survival in GC patients [7], its molecular and functional roles in mediating chemoresistance remain to be explored.

SOX9 is a member of the SRY-related high mobility group box (SOX) transcription factor family [10]. SOX9 regulates diverse cellular processes, including embryonic development, cell fate determination, and maintenance of stem cell properties [11]. Several studies have highlighted the role of SOX9 in promoting cancer stemness where overexpression of SOX9 in a subpopulation of cells within tumors mediates self-renewal capacity, leading to therapeutic resistance, tumor recurrence and poor clinical outcome [12, 13]. Notably, SOX9 marks gastric stem cells and plays a key role in gastric tumor initiation [11].

MicroRNAs (miRNAs) are small non-coding RNAs that post-transcriptionally regulate gene expression by binding to the 3’ untranslated region (3’UTR) of target messenger RNAs (mRNAs), leading to mRNA degradation or translational repression [14]. MiRNAs are deregulated in gastric cancer where they may be tumorigenic or suppressor depending on their expression levels and target gene function [15–18].

This study identifies a CDK1-SOX9-BCL-xL signaling axis as a critical mediator of chemoresistance in gastric cancer. CDK1 stabilizes SOX9 via epigenetic silencing of miR-145, while SOX9 directly upregulates the anti-apoptotic protein BCL-xL, enabling cisplatin evasion. Targeting CDK1 disrupts this pathway, resensitizing resistant cancer cells to therapy and reducing tumor burden in vivo.

## Methods

### Cell culture

AGS cells (American Type Culture Collection, ATCC, Manassas, VA) were cultured in F12 media (GIBCO, Carlsbad, CA). MKN28 and MKN45 cells (Riken Cell Bank, Tsukuba, Japan) were maintained in RPMI 1640 medium. All the media were supplemented with 10% fetal bovine serum (FBS, Invitrogen Life Technologies, Carlsbad, CA) and 1% penicillin/streptomycin (GIBCO) at 37 °C in 5% CO_2_, humidified-cell culture incubator. Short tandem repeat (STR) profiling (Genetica DNA Laboratories, Burlington, NC) was used to authenticate the cell lines. All cell lines tested negative for mycoplasma contamination (R&D Systems, Minneapolis, MN).

### Gastric tumoroids

Patients derived gastric tumoroids were established from PDX498. Briefly, the tissue was sliced into 2–3 mm fragments and treated with 5 mM EDTA for 30 min at 37 °C with agitation. The resulting cell suspension was filtered through 40 μm strainers and centrifuged at 1200 RPM for 5 min. The pellet was mixed with 50 μL of matrigel and seeded into pre-warmed 24-well plates. After polymerization in a CO2 incubator for 20 min, 500 µL of human basal medium was added. After 7–10 days, tumoroids were fixed in 4% paraformaldehyde for 20 min, washed with PBS, and embedded in 30 µL of Histogel. The Histogel-embedded tumoroids were fixed in 70% ethanol. Paraffin-embedded tumoroids were pre-treated at 65 °C for 90 min, deparaffinized, and subjected to antigen retrieval using Tris-EDTA Buffer. Slides were incubated overnight at 4 °C with primary antibodies, then with Alexa Fluor secondary antibodies for 2 h at room temperature. Imaging was performed using a Zeiss LSM980-Airyscan confocal microscope (Hebron, KY).

### Animals

The use of animals was approved by the University of Miami institutional animal care and use committee (IACUC#23–110). We purchased the 129S(B6N)-*Cdk1*^*tm1Eddy*^/J *(Cdk1*^*flox/flox*^) mice and *Krt19*^*Cre/ERT*^ mice from The Jackson Laboratory (Bar Harbor, ME) [19, 20]. The *Cdk1*^*flox/flox*^ and *Krt19*^*Cre/ERT*^ were crossed to obtain the *Krt19*^*CreERT*^*/Cdk1*^*flox/flox*^ mice. These CDK1 conditional knockout mice received Tamoxifen injection (50 mg/ kg) to induce deletion of CDK1, as previously described [7], referred to as *Cdk1*-KO. In addition, we crossed these mice with the *Tff1*^−/−^ [21, 22] to obtain *K19*^*creERT*^ *CDK1*^*flox/flox*^ *Tff1*^*−/−*^, referred to as *CKF.*

The *Tff1*^−/−^ mice (8–10 months age) were used in this study, with ten mice per group. These mice received dinaciclib (20 mg/kg, Selleckchem, Houston, TX, S2768) via intraperitoneal injection three times a week for 4 weeks. The B6.Cg-Prkdcscid/SzJ mice were purchased from The Jackson Laboratory (Bar Harbor, ME). Ten mice per group at the age of 6 weeks were utilized for subcutaneous implantation with a de-identified patient-derived xenograft (PDX539, Supp. Table 4), as previously described [23]. Once the tumors reached 100–150 mm^3^, the mice received treatment. Dinaciclib (20 mg/kg) was administered three times a week for 4 weeks, and cisplatin (1 mg/kg, MedChemExpress, Monmouth Junction, NJ, HY-17394) once a week for 4 weeks, both via intraperitoneal injection. Mice were monitored for survival and euthanized when tumors reached 1000 mm^3^. Frozen and formalin-fixed paraffin-embedded tissue samples were processed for further analysis.

### DNA plasmids and SiRNA transfection

FuGene 4 K (FuGene, 4 K-1000) were used for plasmid transfection and DharmaFECT1 (T-2001-01 (Horizon Discovery Ltd.) were used for siRNA transfection, following the manufacturer’s instructions. ON-TARGETplus human siCTRL (J-005834-05-0005), siCDK1 (L-003224-00-0005) and siSOX9 (L-021507-00-0005) were purchased from Horizon Discovery Ltd. Cdc2-HA was a gift from Sander van den Heuvel (Addgene plasmid #1888) [24]. siDNMT1 (sc-35204) was purchased from Santa Cruz Biotechnology (Dallas, Texas).

### Quantitative reverse-transcriptase real-time polymerase chain reaction (qRT-PCR) analysis

According to the manufacturer’s instruction, total RNA was isolated using TRIzol RNA isolation reagent (Invitrogen, Carlsbad, CA, 15,596,026). For cDNA synthesis, 1 µg of total RNA was reverse transcribed as instructed (Promega, A5000). qRT-PCR was performed using GoTaq 2-Step qRT-PCR System (Promega, A6020). RT-qPCR was carried out using the CFX Connect Real-Time PCR Detection System (Bio-Rad Laboratories, Hercules, CA). The levels of gene transcripts were determined using a 2-ΔCt method and normalized to the HPRT or β-actin. Primers used in PCR are listed in Supp. Table 5.

### Protein analysis and western blotting

Total proteins from monolayer cells were isolated using RIPA lysis buffer with protease and phosphatase inhibitors. Protein concentrations were measured using the Pierce BCA assay kit and a LUMIstar OPTIMA Microplate Luminometer. Proteins were separated by 8–10% SDS-PAGE and transferred to a nitrocellulose membrane. Membranes were blocked with a buffer (0.1% Tween-20, 5% BSA in PBS) for 1 h at room temperature, then probed with a primary antibody overnight at 4 °C. After washing, membranes were incubated with HRP-linked secondary antibodies for 1 h, followed by additional washes. Protein signals were visualized using Luminata Forte substrate and the ChemiDoc MP Imaging system (BioRad).

### Dual luciferase assay

Dual luciferase reporter assay was conducted according to the manufacturer’s instruction (Promega, Madison, WI, E397A). Briefly, cells were seeded in a 6 well tissue culture plate. The next day, cells at 60–70% confluency were transiently transfected with luciferase reporter plasmid (1 µg DNA per well) using FuGene HD transfection reagent as instructed (Promega, E2311). 48 h post-transfection, luciferase reporter gene activity was determined. LUMIstar OPTIMA Microplate Luminometer (BMG LabTech, Cary, NC) was used to measure Relative Luciferase Units (RLUs) in a 96-well plate (Dynex). RLUs were normalized to firefly, and the data were presented as luciferase activity. Each transfection was performed in triplicate.

### Immunohistochemistry analysis

A total of 216 evaluable cases from gastric tissue microarrays (TMAs) that contained cancers along with their corresponding adjacent non-tumor tissues, were utilized for immunohistochemistry (IHC) analysis. IHC staining procedures were done by employing rabbit anti-human CDK1 antibody (ab133327, Abcam, Cambridge, MA) and SOX9 antibody (37447, Cell Signaling, Danvers, MA). Tissue samples were collected, anonymized, and coded in accordance with approved Institutional Review Board protocols. Imaging was conducted using an Olympus FSX100 microscope (Olympus, Japan). Scoring was performed based on predefined criteria, with five randomly selected fields assessed for both intensity and percentage of positively stained cells on each slide. The final scoring for each slide was determined by multiplying the proportion score (0-4) by the intensity score (0-3), resulting in a range of 0 to 12.

### DNA bisulfite treatment and pyrosequencing analysis

DNA was purified using a DNeasy Tissue Kit (Qiagen, Germantown, MD) and bisulfite-modified with an EZ DNA Methylation-Gold Kit (Zymo Research). The miR-145 promoter CpG island was identified using an online search tool (http://www.uscnorris.com). Pyrosequencing primers were designed with PSQ Assay Design Software (Biotage, Vimpelgatan, Sweden), including the forward primer ATGGGGTTGGATGTAGAAGA and the biotin-labeled reverse primer Biotin- TTTCCAAAAATCCCCATCTTAACATCT. A 40-ng sample of modified DNA underwent PCR amplification with the Platinum PCR SuperMix High Fidelity (Invitrogen). PCR products were verified by gel electrophoresis. Quantitative analysis was performed using the Biotage PyroMark MD System, and results were processed with Pyro Q-CpG 1.0.9 software. A cutoff of 10% methylation was set to identify hypermethylation of the miR-145 promoter, and statistical analysis assessed differences in DNA methylation frequencies between the normal and cancer, as well as control and 5-Azacytidine (5ʹ-Aza) treatment.

### Chromatin immunoprecipitation (ChIP)

The chromatin immunoprecipitation (ChIP) assay was performed using Zymo-spin ChIP™ Kit (Zymo research, Irvine, CA, #D5210) according to the manufacturer’s instruction. Purified DNA fragments were analyzed by quantitative PCR (qPCR) using SYBER green master mix (Applied Biosystems, Carlsbad, CA, A46012) using a primer set designed to amplify the ChIP-DNA fragment, consisting of the predicted SOX9 binding sites on the *BCL2L1* promoter. Quantitative PCR was performed according to the manufacturer’s parameters. Supp. Table 5 shows the primer sets used in ChIP-qPCR reactions. The relative occupancy of P1, P2 and P3 was normalized to the IgG signal, and the data were presented as fold enrichment.

### Gene set enrichment analysis (GSEA) and public gene expression data analysis

The reference gene sets were downloaded from the MSigDB database (https://www.gsea-msigdb.org/gsea/index.jsp). Differential expression analysis of genes (DEGs) between CDK1 high and low expression samples was conducted using the ‘limma’ package. The Normalization Enrichment Score (NES) and false discovery rate (FDR) for each gene set were computed using the ‘cluster Profiler’ package (v3.12.0). Gene sets were deemed significant if their FDR was below 25%.

For analysis of significant genes and their expression in gastric cancer, the TCGA gastric cancer cohort was obtained from the National Cancer Institute (NCI) portal (https://portal.gdc.cancer.gov/). Additionally, a series of human gastric cancer datasets were retrieved from the GEO Datasets repository (https://www.ncbi.nlm.nih.gov/gds/). Gene expression was determined using the mean value of probes when a gene had multiple probes. All data analyses were conducted using R software (R 3.6.1; https://www.r-project.org/).

SOX9 gene signatures containing 28 gene sets were obtained by combining 26 SOX9 target gene sets identified from ChIP-seq from 26 stomach-related cell lines from ENCODE [25] and two SOX9-focused gene sets from MSigDB [26]. GSEA analyses were performed using differential express results conducted using TCGA STAD CDK1-high vs. CDK1-low against the SOX9 gene signature gene sets.

### Quantification and statistical analysis

Statistical significance of the in vitro and in vivo studies was analyzed by the one-way ANOVA, 2-tailed Student’s t-test, and Pearson’s Correlation Coefficient analysis using the GraphPad Prism 9 software (GraphPad Software, Inc, San Diego, CA, USA). Significant differences are described in the figure legends as * *P* < 0.05, ** *P* < 0.01 or *** *P* < 0.001.

## Results

### CDK1 and SOX9 are positively correlated in gastric cancer

**Fig. 1:**
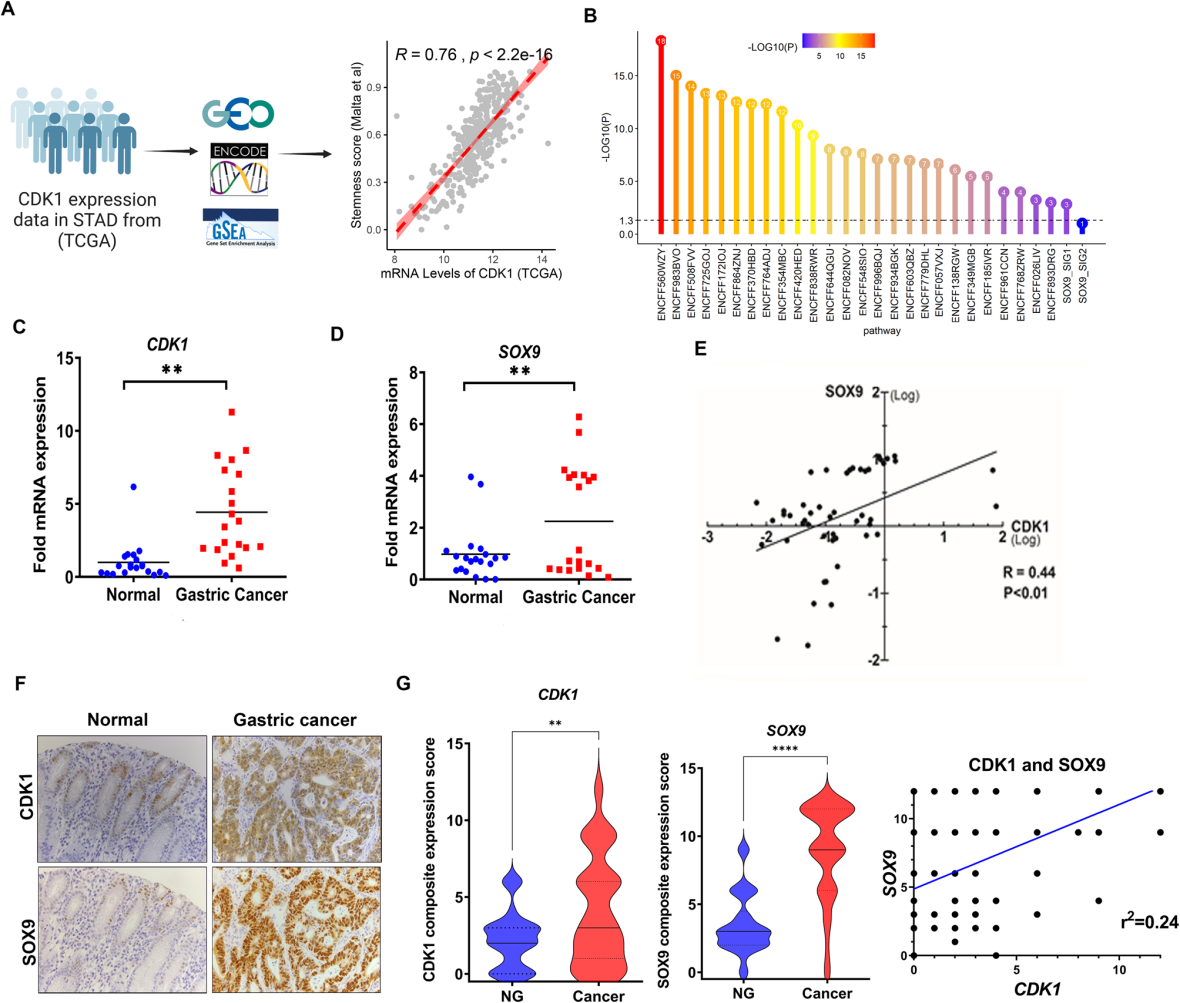
CDK1 and SOX9 are upregulated in gastric cancer. Pearson’s correlation test for *CDK1* mRNA levels from TCGA cohort in GC showed a positive correlation with stemness scores **(A)**. Significant enrichment of differentially expressed genes between *CDK1*-high and *CDK*1-low were observed in 27 of the 28 SOX9 signature gene sets **(B)**. mRNA transcripts of *CDK1* and *SOX9* in human GC samples (*n* = 24) **(C**,** D).** Correlation of *CDK1* and *SOX9* mRNA expression in human gastric samples **(E).** A representative IHC staining of CDK1 and SOX9 in normal and GC tissues **(F)**. Composite expression score of CDK1 and SOX9 in normal (*n* = 108), and GC (*n* = 108) tissues. Spearman’s correlation between IHC staining scores of CDK1 and SOX9 in normal and GC tissues **(G)**. (*P < 0.05, **P < 0.01, ***P < 0.001)

In this study, we investigated the possible association between *CDK1* overexpression and stemness scores to explain the chemoresistance and poor clinical outcomes. We used the TCGA dataset and stemness scores associated with oncogenic de-differentiation [27]. This analysis revealed a positive correlation between *CDK1* expression and stemness scores in GC (Fig. 1A, *P* < 0.001). Because SOX9 functions as a pivotal factor in modulating stem cell transformation, thereby promoting gastric tumorigenesis [28], we investigated whether *CDK1* expression correlates with SOX9 transcription signature in patients with gastric cancer. 27 out of 28 SOX9 signature gene sets showed a significant enrichment of differentially expressed SOX9 target genes between *CDK1*-high and *CDK1*-low (Fig. 1B). We also detected significant association of high *CDK1* and *SOX9* transcripts in the TCGA dataset (*P* < 0.01) (supp Fig. 1A and supp. Figure 1B). Confirming these findings, we detected a positive correlation between high *CDK1* expression and SOX9 target genes’ signature levels (supp Fig. 1C, SOX9_B1 [29], NES: 1.56, FDR q value: 0.01 and Schaeffer SOX9 targets [30], NES: 1.67, FDR q value: 0.09, respectively). *CDK1* and *SOX9* transcripts were high in our local data set of de-identified human gastric cancer tissue samples (Fig. 1C and D), and murine gastric cancer samples (supp. Figure 1D), with a notable positive correlation (Fig. 1E and supp. Figure 1D). Immunohistochemistry (IHC) staining confirmed these results, demonstrating a significant correlation between CDK1 and SOX9 protein levels in de-identified human GC tissue samples (Fig. 1F and G, *P* < 0.01). Using Kaplan-Meier survival curve analysis, we found that co-expression of CDK1 and SOX9 was linked to decreased overall survival in high-risk gastric cancer patients (supp. Figure 1E). These findings alluded to a potential functional relationship between CDK1 and SOX9, suggesting a role in driving gastric tumorigenesis and possibly chemoresistance.

### CDK1 mediates an increase in SOX9 protein and activity levels

**Fig. 2:**
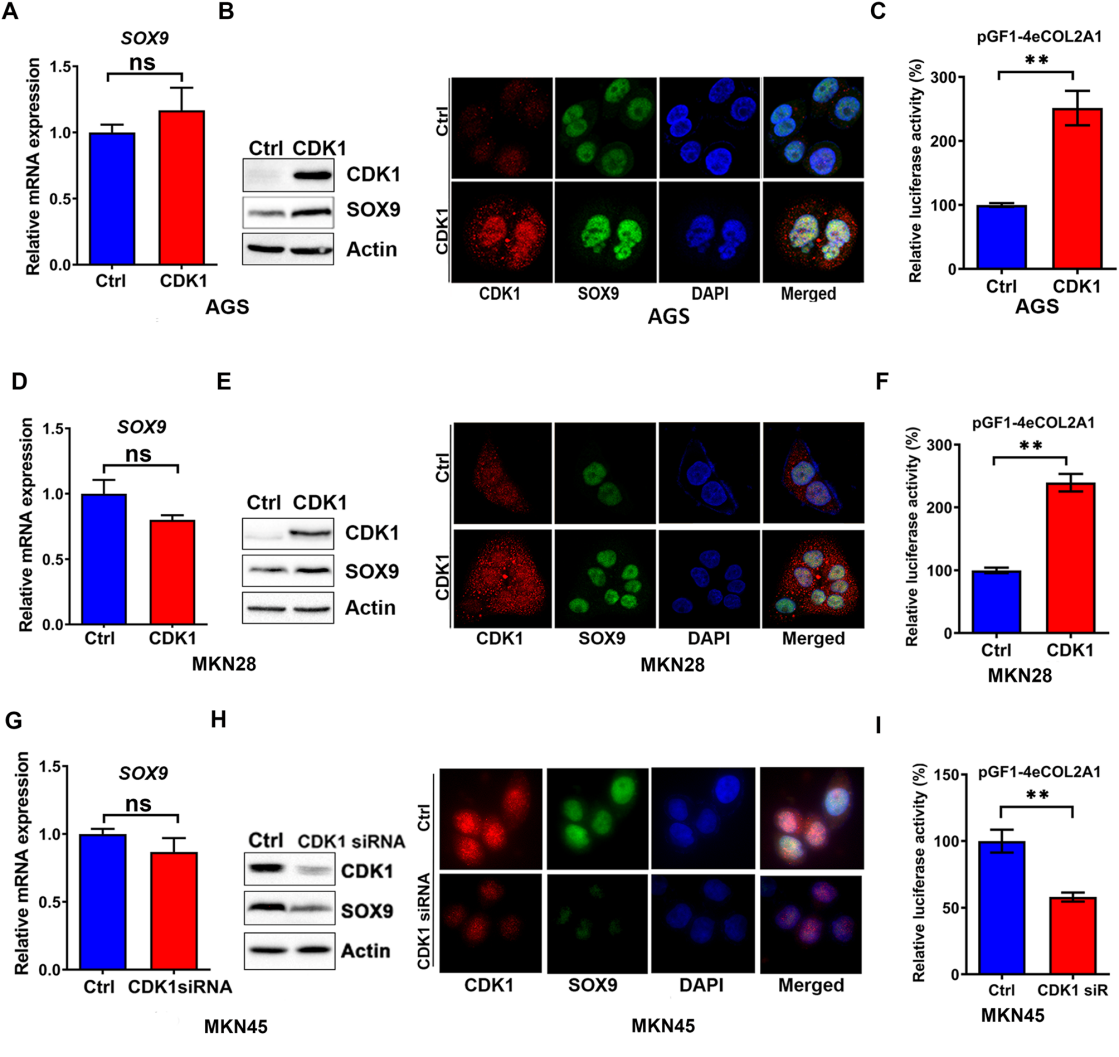
CDK1 activates SOX9 in gastric cancer. Ectopic overexpression of CDK1 or empty vector (Ctrl) for 72 h in AGS cells had no effect on *SOX9* mRNA expression **(A)**. While remarkably increasing the SOX9 protein level by western blot analysis (left panel) and increase in nuclear SOX9 following overexpression of CDK1 was detected by Immunofluorescence analysis (right panel) **(B)**. This was associated with a significant increase in SOX9 luciferase reporter activity **(C)**. Similar findings were observed in MKN28 cells **(D-F).** The knockdown of CDK1 in MKN45 cells had no effect on *SOX9* mRNA **(G).** Western blot analysis and immunofluorescence analysis show a decrease in nuclear SOX9 following knockdown of CDK1 in MKN45 cells **(H)**. This was associated with a significant decrease in SOX9 luciferase reporter activity **(I)**. The firefly luciferase activity was normalized to renilla luciferase activity. scale bar = 20 μm

To investigate the role of CDK1 in regulating SOX9, we overexpressed CDK1 in AGS and MKN28 cells with relatively low CDK1 expression and silenced CDK1 in MKN45 cells with constitutively high CDK1 expression. Ectopic CDK1 expression did not affect *SOX9* transcript levels in AGS (Fig. 2A) and MKN28 cells (Fig. 2D). Similarly, the knockdown of CDK1 had no effect on *SOX9* mRNA expression in MKN45 cells (Fig. 2G). However, the overexpression of CDK1 induced significant increase in SOX9 protein levels, as shown in western blot and immunofluorescence analysis in AGS (Fig. 2B) and MKN28 cells (Fig. 2E), while silencing of CDK1 reversed these effects in MKN45 cells (Fig. 2H).

Using the SOX9 luciferase reporter assay that contains *COL2A1* gene promoter with SOX9 binding sites, as a measure of SOX9 transcription activity, we detected significant increase in the luciferase activity following CDK1 overexpression in AGS (Fig. 2C, *P* < 0.01) and MKN28 cells (Fig. 2F, *P* < 0.01). Conversely, the knockdown of CDK1 in MKN45 cells showed opposite effects (Fig. 2I, *P* < 0.01). Taken together, these findings indicate that CDK1 induces SOX9 protein and transcription activity levels in GC cells.

### miR-145 regulates SOX9 via a CDK1-dependent mechanism

**Fig. 3:**
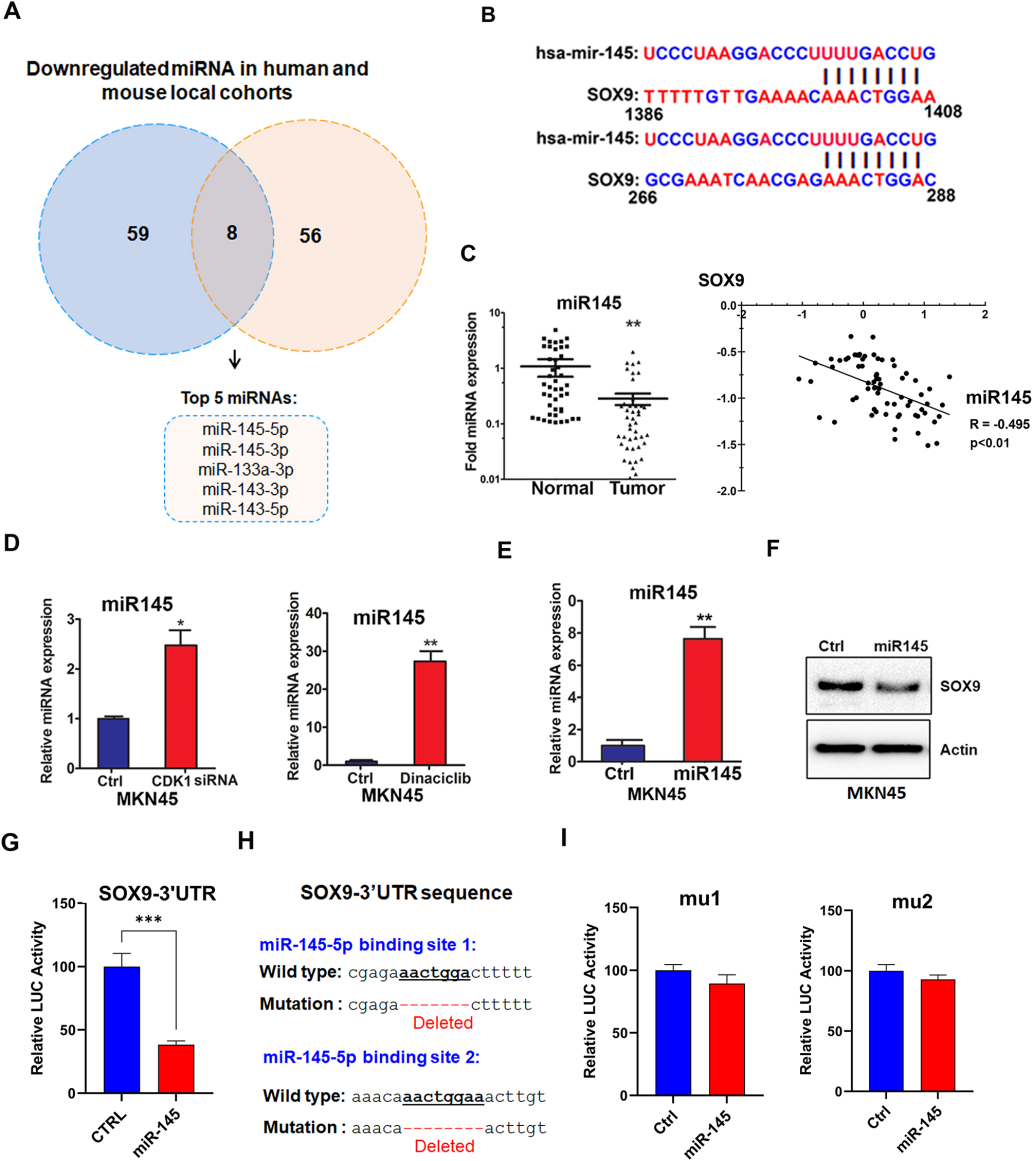
CDK1 regulates SOX9 levels via miR-145. A Venn diagram showing downregulated miRNAs in human (blue) and mouse (orange) local cohorts **(A).** miR-145 putative binding sites on the 3’UTR of *SOX9* are shown **(B).** miR-145 expression in human normal and GC tissue samples, *P* < 0.01. A negative correlation between *SOX9* and miR-145 expression levels is detected in human gastric samples **(C)**. MKN45 cells were treated with dinaciclib (20nM) for 16 h or transfected with siCDK1 or siCtrl for 72 h then qRT-PCR conducted to measure the expression levels of miR-145 **(D).** MKN45 cells were transfected with miR-145 or the empty vector (Ctrl) for 72 h, miR-145 levels were determined by qRT-PCR **(E)** and SOX9 protein levels were detected by western blot analysis. A representative β-actin is shown as an internal control in WB **(F).** AGS cells were transfected with the SOX9-3’UTR reporter and Rluc luciferase constructs with an empty vector (Ctrl) or miR-145 **(G)**, SOX9-3’UTR mu1, or SOX9-3’UTR mu2 **(H)**. After 48 h of transfection, firefly luciferase activity in the cell lysates was measured, and reporter activity was normalized to renilla luciferase activity as shown **(I)**

As shown in Fig. 2, CDK1 has no effect on the mRNA expression of *SOX9* in gastric cancer, indicating a possible post-transcription regulatory mechanism. We hypothesized a possible role of miRNAs in regulating SOX9. Using comprehensive miRNA sequencing of gastric tumors from murine and de-identified human tissues [18] demonstrated conserved downregulation of miRNAs (Fig. 3A). Integrated bioinformatics analysis approach (TargetScanHuman 7.1) indicated the presence of two putative binding sites for miR-145 within the 3’UTR of SOX9 (Fig. 3B). qRT-PCR analysis of 48 samples of normal and gastric cancer tissues confirmed the significant downregulation of miR-145 in both human and mouse GC (Fig. 3C and supp. Figure 2A, *P* < 0.01). Furthermore, *SOX9* and miR-145 expression levels were negatively correlated (Fig. 3C, *R*=-0.495). These data suggest a possible role of miR-145 in regulating SOX9.

We next investigated whether CDK1 affects miR-145 levels by using CDK1 genetic knockdown or pharmacologic inhibition with dinaciclib [31] in MKN45 cells. The results demonstrated a notable increase in miR-145 expression under these conditions (Fig. 3D, *P* < 0.05 and *P* < 0.01, respectively). Overexpression of miR-145 decreased SOX9 protein levels (Fig. 3E and F and supp. Figure 2B and 2C), confirming the link between miR-145 and SOX9. To confirm the role of miR-145, we used a SOX9-3’UTR reporter that contain the putative binding sites of miR-145. Overexpression of miR-145 decreased the luciferase activity (Fig. 3G, *P* < 0.001). To further verify the functionality of the binding sites, we developed a reporter with mutations in these binding sites (mu1 and mu2, Fig. 3H). The results confirmed the importance of these sites, showing no change in the mutated SOX9-3’UTR luciferase reporter activity following miR-145 overexpression (Fig. 3I). Collectively, these findings indicate that miR-145, suppressed by CDK1, plays a critical role in regulating SOX9 levels in gastric cancer cells.

### CDK1 phosphorylates and activates DNMT1 to mediate methylation and suppression of miR-145 expression

**Fig. 4:**
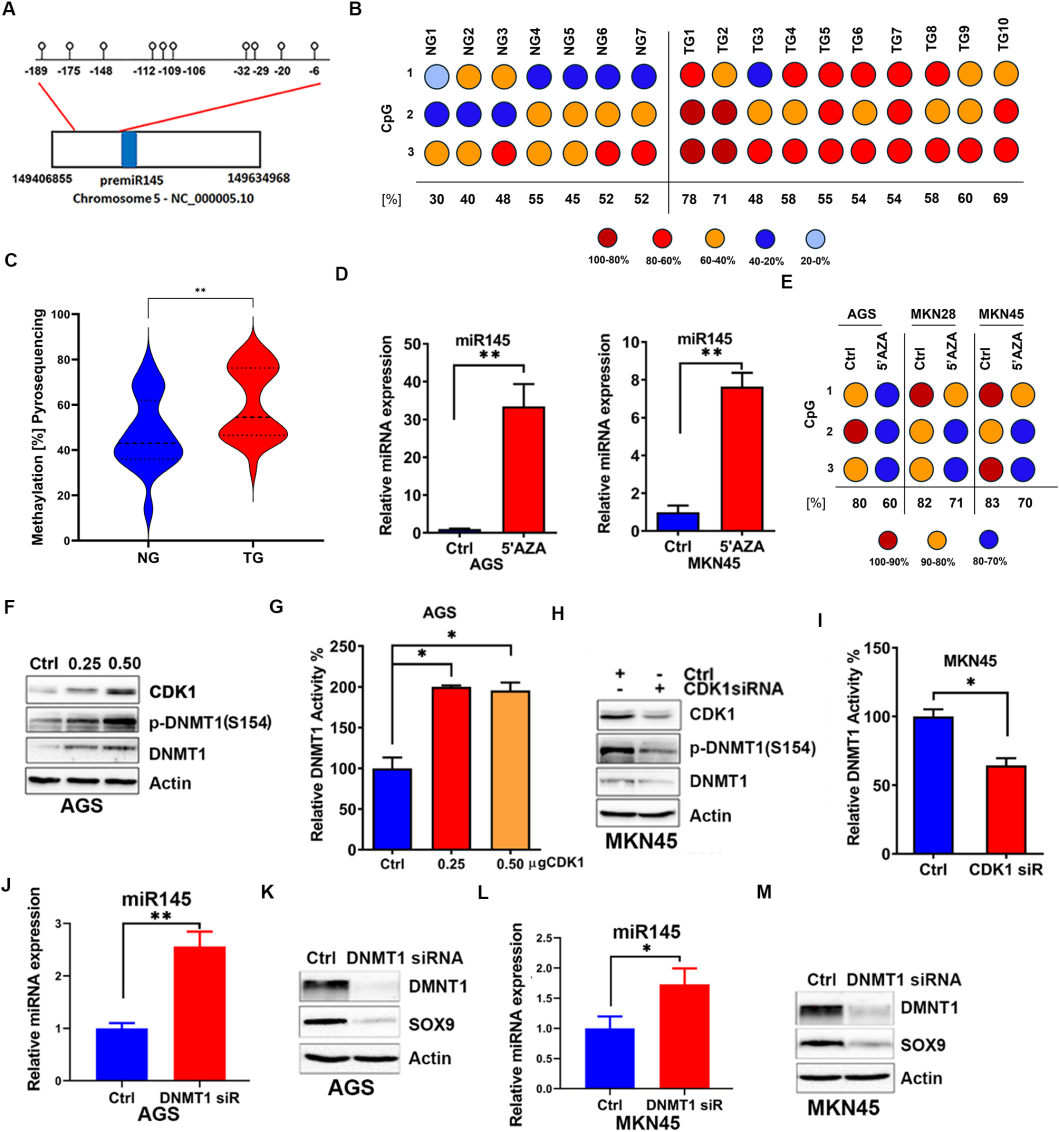
CDK1 suppresses miR-145 expression via induction of DNMT1. The miR-145 promoter shows a CpG island with several CpG nucleotides **(A).** Representative dot plots of methylation status of CpG sites in normal (NG = 7) and tumor (TG = 10) gastric human samples detected by pyrosequencing assay **(B).** Methylation levels of miR-145 in normal and tumor human gastric samples **(C).** qRT-PCR analysis shows an increase miR-145 expression in AGS and MKN45, following treatment with 5’Aza for 48 h **(D).** Representative dot plots of methylation status of CpG sites in control and 5’Aza treated GC cells detected by pyrosequencing assay (**E**). Overexpression of CDK1 (0.25 and 0.5 µg), increases total and pDNMT1 (S145) levels, as compared to control, a representative ß-actin used as an internal control is shown **(F)**. CDK1 knockdown (siCDK1) showed the opposite effects, as compared to control (siCtrl) **(H).** DNMT1 enzymatic activity increases following CDK1 overexpression (**G**), with a reverse effect following CDK1 knockdown **(I)**. Knockdown of DNMT1 in AGS and MKN45 cells led to an increase in miR-145 and decrease SOX9 protein expression (**J - M**)

To investigate the mechanism by which CDK1 regulates SOX9 protein expression through miR-145, we analyzed the premiR-145 and identified a CpG island with several CpG nucleotide sites in the miR-145 promoter (Fig. 4A). Based on these findings, we hypothesized that methylation plays a role in regulating the expression of miR-145. We investigated miR-145 promoter methylation levels in normal and tumor gastric patients by methylation-sensitive pyrosequencing. Cancer tissues showed higher levels of methylation compared to the normal (Fig. 4B and C, *P* < 0.01). Indeed, treatment of AGS and MKN45 cell lines with a DNMT inhibitor (5’Aza) led to an increase in miR-145 expression (Fig. 4D, *P* < 0.01). Also, we analyzed the CpG sites in GC cell lines after 5’Aza treatment and the results showed decreased methylation in treated cells compared to control (Fig. 4E). Notably, we detected a CDK1-dependent increase in DNMT1 protein and phosphorylation levels. Overexpression of CDK1 increased DNMT1 levels (Fig. 4F) whereas its knockdown reversed this effect (Fig. 4H). The enzymatic activity of DNMT1 was consistent with the observed changes in DNMT1 levels (Fig. 4G and I). Additionally, the knockdown of DNMT1 in AGS and MKN45 cells induced miR-145 expression, consistent with the 5’Aza results (Fig. 4J − 4 M). Furthermore, treatment with 5’Aza similarly decreased SOX9 protein expression, as shown in supp. Figure 2D. Taken together, our findings demonstrate a novel role for CDK1 in inducing SOX9 levels through DNMT1-mediated inhibition of miR-145.

### SOX9 induces BCL-xL expression and activity

**Fig. 5:**
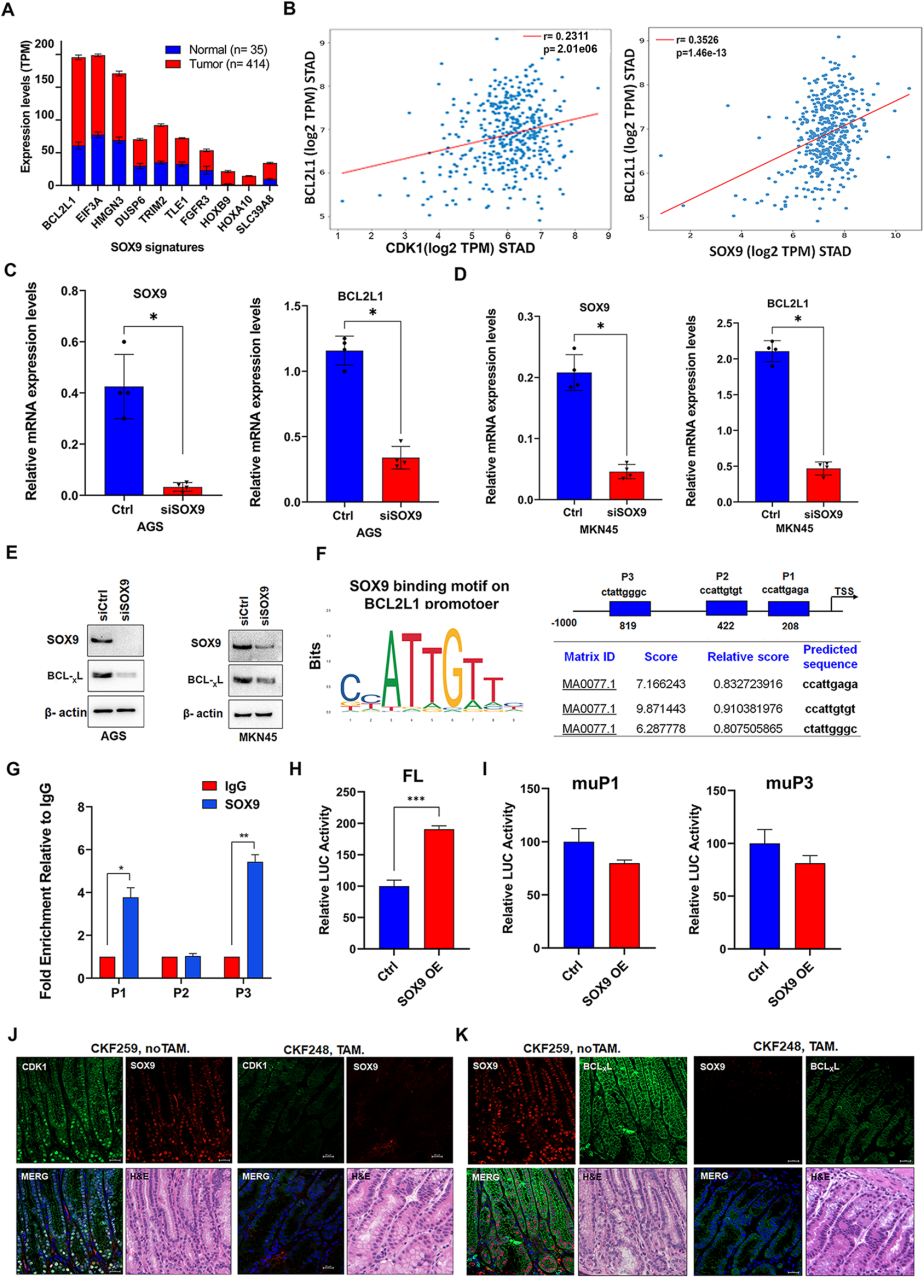
*BCL2L1* is a direct transcription target of SOX9. Graph illustrating the expression level of the top 10 SOX9 signature genes in the TCGA STAD dataset **(A).** A positive correlation was observed between the expression levels of *CDK1* and *BCL2L1* (BCL-xL), and between *SOX9* and *BCL2L1* in the STAD dataset **(B)**. qRT-PCR analysis of *BCL2L1* mRNA expression, following SOX9 knockdown (siSOX9) or control (siCtrl) in AGS **(C)** and MKN45 cells **(D)**. Subsequent western blot analysis demonstrates a decrease in BCL-xL protein, following SOX9 knockdown **(E)**. Predicted SOX9 binding sites motif within the *BCL2L1* promoter were identified using the JASPAR database **(F)**. ChIP-qPCR analysis demonstrates enrichment of P1 and P3 binding sites, normalized to the IgG control **(G)**. AGS cells were transfected with SOX9 plasmid and *BCL2L1* full length promoter (FL) reporter, along with luciferase reporter plasmids containing mutated SOX9 binding sites (muP1 and muP3) shows a decrease in the luciferase reporter activity with SOX9 mutations. Firefly luciferase activity was measured after 48 h of transfection, and reporter activity was normalized to renilla luciferase activity **(H**,** I)**. *Krt19*^*Cre*^*/Cdk1*^*flox/flox*^ / *Tff1*^*−/−*^ mice tissues were proceeded for immunofluorescence staining of CDK1, SOX9 and BCL_X_L. DAPI is used for nuclear staining **(J**,** K)**. scale bar = 20 μm

To investigate the functional impact of CDK1-SOX9 axis, we first analyzed publicly available datasets comprising two distinct studies focused on SOX9 signature genes [29, 30]. This was followed by evaluating the SOX9 target genes’ expression in the gastric cancer TCGA dataset (STAD). We found that the *BCL2L1* gene (encoding the BCL-xL protein) is one of the top five genes on the list (Fig. 5A). In addition, we found that *BCL2L1* positively correlated with *CDK1* and *SOX9* expression (Fig. 5B, *P* < 0.001), suggesting that *BCL2L1* is a downstream target of the CDK1-SOX9 axis in GC. The knockdown of SOX9 resulted in a reduction in *BCL2L1* mRNA and protein (BCL-xL) in both AGS and MKN45 cell lines (Fig. 5C and E).

We next investigated whether *BCL2L1* promoter contains binding sites for SOX9. Using the JASPAR database [32] we detected multiple putative SOX9 binding on the *BCL2L1* promoter. We selected the binding site motif sequence with the highest prediction score for experimental validation (Fig. 5F). Using chromatin immunoprecipitation (ChIP) assay, we detected the binding of SOX9 on two of the three predicted sites in the *BCL2L1* promoter in AGS cells (Fig. 5G). To verify the functionality of these binding sites, we developed a *BCL2L1* promoter luciferase reporter containing the SOX9 binding sites. Indeed, SOX9 overexpression increased the *BCL2L1* promoter activity, whereas mutation of the SOX9 binding sites abrogated these effects (Fig. 5H and I, *P* < 0.001). Collectively, these findings elucidate the binding of SOX9 on the *BCL2L1* promoter to induce its transcription.

### In vivo models confirm the role of CDK1 in regulating the SOX9-BCL-xL axis

We utilized *Tff1*^*−/−*^ mice, which develop low-grade dysplasia (LGD), high-grade dysplasia (HGD), and invasive adenocarcinoma [22]. This model develops gastric tumors exclusively in the antropyloric region [21, 22]. We assessed CDK1 and SOX9 expression in wild-type (WT) controls and *Tff1*^*−/−*^ mice with high-grade dysplasia (HGD), and intramucosal carcinoma (IMC) (supp. Table 2). Both CDK1 and SOX9 levels were significantly elevated in HGD and IMC compared to WT (supp. Figure 3A) supporting the involvement of an intact CDK1-SOX9 axis in gastric tumorigenesis. We then treated these mice with the CDK1 inhibitor dinaciclib for four weeks. Dinaciclib treatment mitigated this elevation as demonstrated by western blot analysis (supp. Figure 3B). This was further confirmed by immunofluorescence analysis (supp Fig. 3C and D).

To genetically verify the pharmacologic approach, we bred the *Tff1*^*−/−*^ mouse with the *Krt19*^*CreERT*^*/Cdk1*^*flox/flox*^ [7] to develop the *Krt19*^*CreERT*^*/Cdk1*^*flox/flox*^ /*Tff1*^*−/−*^, and we referred to CKF (supp. Table 3). The conditional knockout of CDK1 in TFF1^−/−^ mice markedly diminished the advancement of histological abnormalities, leading to reduced occurrence of high-grade dysplasia and adenocarcinoma (supp Fig. 4A and B). Tamoxifen treatment of these mice decreased levels of CDK1, SOX9, and BCL-xL proteins, compared to the untreated mice (Fig. 5J and K). These results confirm the integrity of the CDK1-SOX9-BCL-xL axis in vivo.

### Dinaciclib suppresses SOX9-BCL-xL and reverses chemotherapeutic resistance axis

**Fig. 6:**
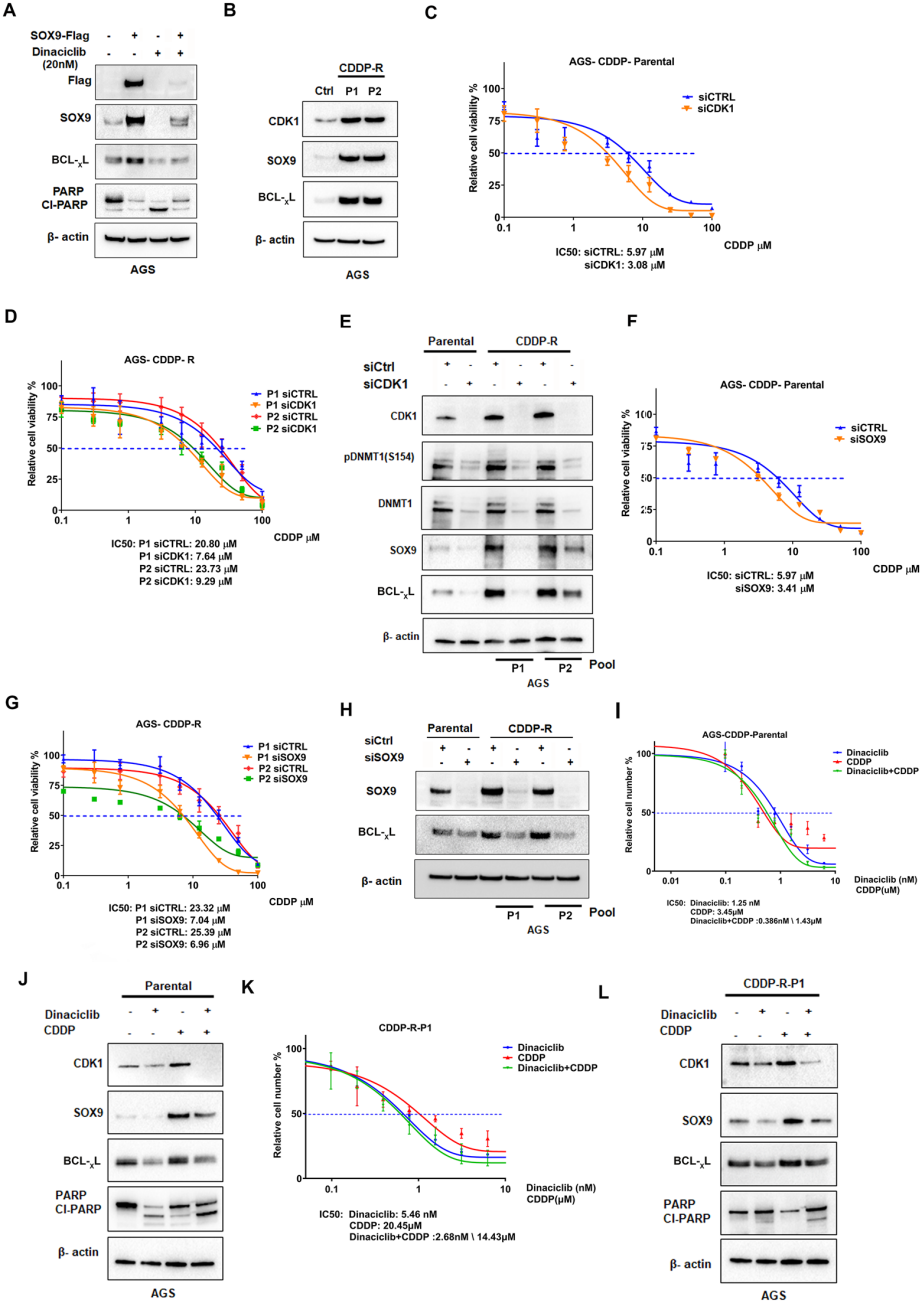
Silencing CDK1 and SOX9 sensitizes AGS cells to CDDP treatment. AGS cells were transfected with SOX9, or empty vector (Ctrl) then treated with 20nM dinaciclib for 16 h. Whole cell lysate (WCL) was collected and subjected to western blot analysis, membranes were probed with CDK1, SOX9, BCL-_X_L, PARP, Cl-PARP and β-actin **(A).** WCL of AGS parental cells and CDDP-R cells (P1 and P2) were collected and subjected to western blot, membranes were probed with CDK1, SOX9, BCL-_X_L, and β-actin **(B)**. AGS parental cells and CDDP-R cells (P1, and P2) were transfected with siCtrl or siCDK1 for 72 h and IC50 was performed using ATP-GLO as instructed **(C**,** D)**. AGS parental cells and CDDP-R cells (P1 and P2) were transfected with siCDK1 or siCtrl for 72 h, WCL were collected and subjected to western blot. Membranes were probed with CDK1, p-DNMT1 (S154), SOX9, BCL-_X_L, and β-actin **(E)**. AGS parental cells and CDDP-R cells (P1, and P2) were transfected with siCtrl, siSOX9 for 72 h and IC50 was performed using ATP-GLO as instructed **(F**,** G)**. AGS parental cells and CDDP-R cells (P1 and P2) were transfected with siSOX9 or siCtrl for 72 h, WCL were collected and subjected to western blot analysis, membranes were probed with CDK1, SOX9, BCL-_X_L, and β-actin **(H)**. AGS parental cells and CDDP-R cells were treated with cisplatin and dinaciclib, either alone or in combination. Cell viability was then assessed using the ATP-Glo assay to determine the IC50 **(I**,** K)**. AGS parental cells and CDDP-R cells (P1) were treated with cisplatin 10 μm and dinaciclib 20nM, WCL was collected and subjected to western blot. Membranes were probed with CDK1, SOX9, BCL-_X_L, PARP, Cl-PARP, and β-actin **(J**,** L)**

Chemotherapeutic resistance is a significant challenge in the treatment of GC patients, leading to treatment failure and poor clinical outcomes. To determine the role of CDK1-SOX9 axis and the importance of SOX9 in the process, we performed an experiment using CDK1 inhibitor (dinaciclib) alone and with SOX9 rescue (overexpression) in AGS cells. Dinaciclib treatment led to a decrease in SOX9 and BCL-xL along with an increase in cleaved PARP (Cl-PARP), an apoptotic marker. Remarkably, the overexpression of SOX9 in the presence of dinaciclib was associated with a decrease in Cl-PARP levels, confirming the protective effect of SOX9 against apoptosis (Fig. 6A). To further confirm the role of CDK1-SOX9 axis in resistance, we developed AGS cisplatin-resistant cells (CDDP-R) (Pool1 and Pool2). Western blot analysis showed elevated protein levels of CDK1, SOX9, and BCL-_X_L in CDDP-R cells, as compared to parental control cells (Ctrl) (Fig. 6B). To investigate the role of CDK1 and SOX9 in CDDP-R cells, we knocked down CDK1 in AGS parental and CDDP-R cells. The results of IC50 assays demonstrated that CDK1 knockdown re-sensitized CDDP-R cells to cisplatin (Fig. 6C and D). Also, silencing CDK1 led to reduced levels of pDNMT1, SOX9 protein and its downstream target BCL-_X_L (Fig. 6E). Similarly, silencing of SOX9 using siSOX9 resensitized CDDP-R cells to cisplatin (Fig. 6F and G) and resulted in decreased SOX9 protein levels and BCL-_X_L expression (Fig. 6H). Additionally, we confirmed this observation utilizing dinaciclib and cisplatin (CDDP) treatment. The combination of dinaciclib and CDDP decreased the viability of the cells compared to the dinaciclib and CDDP alone in both parental and CDDP-R cells (Fig. 6I and K). The protein levels of CDK1, pDNMT1, SOX9 and BCL-_X_L were reduced with the treatment combination (Fig. 6J and L, supp Fig. 5A and B). Collectively, our findings provide evidence that the CDK1-SOX9 axis contributes to chemotherapy resistance in GC, highlighting its potential as a therapeutic target in overcoming drug resistance.

**Fig. 7:**
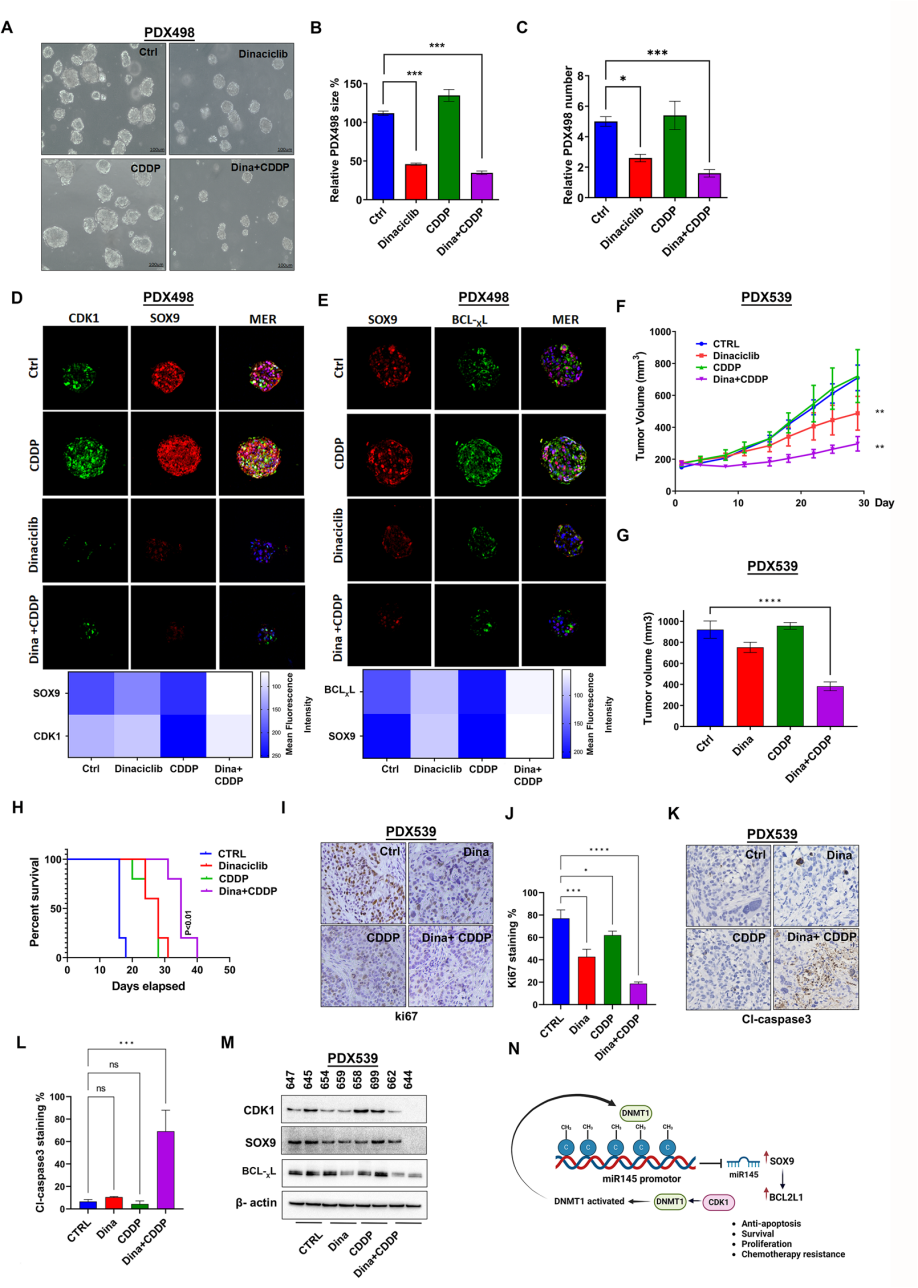
CDK1 and SOX9 driving tumorigenesis in vitro and in vivo. A representative image of the tumoroids. Tumoroids were generated from patient-derived tumor xenografts (PDX498) then treated with Dinaciclib (10 nm), CDDP (10 μm) or combination for 72 h. scale bar = 100 μm. **(A).** A quantification of tumoroids size **(B)** and tumoroids number **(C)** for each condition, 5 random locations were measured and quantified using ImageJ. Tumoroids were collected and fixed then proceeded for immunofluorescence staining of CDK1 and SOX9 **(D)**, SOX9 and BCL-_X_L **(E)**. DAPI is used for nuclear staining and staining intensity was calculated using imageJ. scale bar = 20 μm. The tumor growth curve for PDX539 implanted in mice that are treated with Dinaciclib (20 mg/kg), CDDP (1 mg/kg), or combination **(F)**. Tumor width and length were measured two times weekly to calculate volume **(G)**. Kaplan-Meier survival curve **(H)**. PDX539 implanted in mice were treated with dinaciclib and cisplatin, tissues were collected and proceeded for immunohistochemistry (IHC) staining of Ki67 **(I)** and Cl-Caspase3 **(K)**. The staining intensity was calculated using imageJ (**J**,** L**). WCL was collected from the tissues and subjected to western blot analysis, membranes were probed with CDK1, SOX9 and BCL-_X_L, and β-actin **(M)**. A cartoon illustrating summary of the results is shown in panel **(N)**

To further confirm the role of CDK1 and SOX9 in driving chemotherapeutic resistance, we employed gastric cancer tumoroids derived from de-identified patient-derived xenograft (PDX498). Initially, we subjected the tumoroids to a 72-hour treatment with dinaciclib, CDDP, and a combination of both drugs. Interestingly, while CDDP treatment led to a slight increase in tumoroids size, treatment with dinaciclib alone or the combination of dinaciclib and CDDP resulted in a reduction in tumoroid size (Fig. 7A and B, *P* < 0.001). Moreover, treatment with dinaciclib alone or in combination with CDDP decreased the number of tumoroids (Fig. 7C, *P* < 0.001). Immunofluorescence staining revealed elevated protein levels of CDK1, SOX9, and BCL-_X_L in surviving CDDP-treated tumoroids, confirming activation of these proteins in treatment resistance, whereas treatment with dinaciclib alone or in combination with CDDP reduced these protein levels (Fig. 7D and E). Next, we explored the effects of dinaciclib alone and in combination with CDDP in vivo for four weeks, utilizing PDX-engrafted mice (PDX539). The results revealed that the growth curve and the tumor volume were significantly decreased with the combination treatment compared to CDDP or dinaciclib alone (Fig. 7F and G). The combination treatment increased the overall survival of mice, compared to CDDP or dinaciclib alone (Fig. 7H). Additionally, the expression of Ki-67, a proliferative marker, showed a significant decrease in the combination treatment group (Fig. 7I and J). The cleaved caspase3 (Cl-caspase3) expression, an apoptosis marker, was significantly increased in the combination treatment group (Fig. 7K and L). Western blot analysis using tumors from the groups demonstrated a notable decrease in CDK1, SOX9 and BCL- _X_L protein levels in the combination group (Fig. 7M). Figure 7N features a cartoon that summarizes the results. Altogether, the in vivo efficacy data elucidate a novel therapeutic concept of a combination of dinaciclib and CDDP for overcoming drug resistance.

## Discussion

Gastric cancer ranks among the most prevalent lethal malignancies worldwide [33]. Treatment refractory cells (TRCs), enriched for stemness properties [34], are a major cause of treatment failure. In contrast to the common dynamic behavior of actively proliferating tumor cells, TRCs can survive and expand under treatment and resurge into the cell cycle at a slow rate [35, 36]. In this study we report the role of CDK1 in mediating gastric cancer chemoresistance by activating SOX9 via an epigenetic mechanism. CDK1 mediates SOX9 activity enabling cells to expand and escape apoptosis via induction of antiapoptotic protein, BCL-_X_L in vitro and in vivo. The expression of CDK1 correlates with poor prognosis and aggressive tumor behavior [37], highlighting its potential as a therapeutic target for overcoming chemoresistance in gastric cancer.

SOX9, a transcription factor crucial in embryonic development and cell fate determination, plays a major role in gastric tumorigenesis [38, 39]. SOX9 is recognized as a marker of cancer stem-like cells, with its expression correlating with poor prognosis and aggressive tumor behavior [28, 37]. Our study uncovers a mechanism by which CDK1 contributes to chemoresistance in GC through its non-canonical function in epigenetically regulating SOX9. Intriguingly, while altering CDK1 expression did not affect *SOX9* transcript levels, it significantly impacted SOX9 protein levels and transcription activity, alluding to a post-transcriptional regulation.

MicroRNAs (miRNAs) regulate several cancer genes and pathways in context-dependent and tissue-specific manners [14]. Our results identify a novel mechanism of regulation of SOX9 in gastric cancer via an epigenetic machinery that includes CDK1-mediated activation of DNMT1 and downregulation of miR-145. This finding adds to the complexity of the context dependent roles of miRNAs where different miRNAs regulate SOX9 in other cancer types such as miR-124 [40] and miR-32 [16] in non-small cell lung cancer [16, 40] and miR-134-2p in breast cancer [41].

TFF1-deficient (TFF1^−/−^) mice consistently develop spontaneous gastric tumors, mainly in antrum of the stomach [22], recapitulating the stepwise progression from hyperplasia to invasive adenocarcinoma seen in human gastric cancer. Loss of TFF1 leads to chronic inflammation and activation of oncogenic pathways like NF-κB and STAT3, which drive tumorigenesis [21, 42]. Conditional deletion of CDK1 in these mice does not prevent lesion formation but significantly reduces the severity and frequency of high-grade dysplasia and invasive cancer, resulting in a less aggressive disease course (supp Fig. 4A and B). Using the *Tff1*^−/−^ mouse model of gastric tumorigenesis [21], our data revealed elevated levels of CDK1, SOX9, and BCL-xL proteins in mice exhibiting high-grade dysplasia and adenocarcinomas. The use of tamoxifen to induce Cdk1 deletion in the *Krt19*^*CreERT*^*/Cdk1*^*flox/flox*^/*Tff1*^*−/−*^ mouse model showed a decrease in SOX9 and BCL-xL expression, confirming the important role of CDK1 in activation of SOX9-BCL-xL signaling axis in vivo. We identified BCL-xL as a novel direct transcription target of SOX9 in gastric cancer. BCL-xL is a critical anti-apoptotic protein that plays a key role in cell survival and resistance to cell death by suppressing pro-apoptotic proteins, including BAX and BAK [43]. Tumoroids are known to be enriched for stem cell properties and largely resistant to therapy [44]. Upon treating the tumoroids from gastric cancer with dinaciclib, cisplatin, and a combination of both drugs, we observed that both dinaciclib alone and the combination with cisplatin significantly reduced the size and number of tumoroids and the protein levels of CDK1, SOX9, and BCL-xL in this model. Dinaciclib is a relatively new selective cyclin-dependent kinase (CDK) inhibitor, including CDK1, that is undergoing testing in several clinical trials [45, 46]. Consistent with these findings, we detected an intact CDK1-SOX9-BCL-xL axis in cisplatin resistant cells where its inhibition or silencing sensitized the cancer cells to cisplatin treatment. We confirmed our findings using a PDX model which recapitulated the results seen in cell lines and tumoroids, demonstrating a significant increase in mice survival with the combination of cisplatin and dinaciclib.

## Conclusions

Our study emphasizes the significant role of CDK1 in chemotherapy resistance in gastric cancer. We demonstrated a novel signaling axis whereby CDK1 activates SOX9 and mediates resistance through activation of SOX9-BCL-xL via an epigenetic mechanism involving downregulation of miR-145. Our findings suggest that targeting the CDK1-SOX9 axis using dinaciclib can offer a potential therapeutic strategy for improving gastric cancer patients’ response to therapy and clinical outcome.

## Supplementary Information

Below is the link to the electronic supplementary material.

Supplementary Material 1

Supplementary Material 2

## Data Availability

All data generated or analyzed during this study are included in this published article and its supplementary information files. The data of RNA expression profiles and clinical information of gastric cancer have been downloaded from The Cancer Genome Atlas (TCGA) official website (https://portal.gdc.cancer.gov/repository). The Gene Expression Omnibus (GEO) datasets were retrieved from National Center for Biotechnology Information (NCBI) GEO database (https://www.ncbi.nlm.nih.gov/).

## Declarations

### Provenance and peer review

Not commissioned, externally peer reviewed.

## Abbreviations

CDK1: Cyclin dependent kinase 1
SOX9: SRY-box transcription factor 9
BCL-xL: B-cell lymphoma-extra-large
TFF1: Trefoil factor 1 (also known as pS2)
BCL2L1: BCL2 like 1
Krt19/K19: Keratin 19
BAX: BCL2-associated X
BAK: BCL2 antagonist/killer 1
ChIP: Chromatin Immunoprecipitation
miR: MicroRNA
qRT-PCR: Quantitative real-time polymerase chain reaction
WB: Western blot
LGD: Low-grade dysplasia
HGD: High-grade dysplasia
CA: Carcinoma
PDX: Patient-derived xenograft
