# Supplementary material for: CDK1 drives SOX9-mediated chemotherapeutic resistance in gastric cancer
Source: J Exp Clin Cancer Res. 2025 Oct 8;44:284. doi: 10.1186/s13046-025-03523-3 (PMC12506374; doi:10.1186/s13046-025-03523-3)
Supplement: Supplementary file 1 — Supplementary Material 1 [file 13046_2025_3523_MOESM1_ESM.pdf]

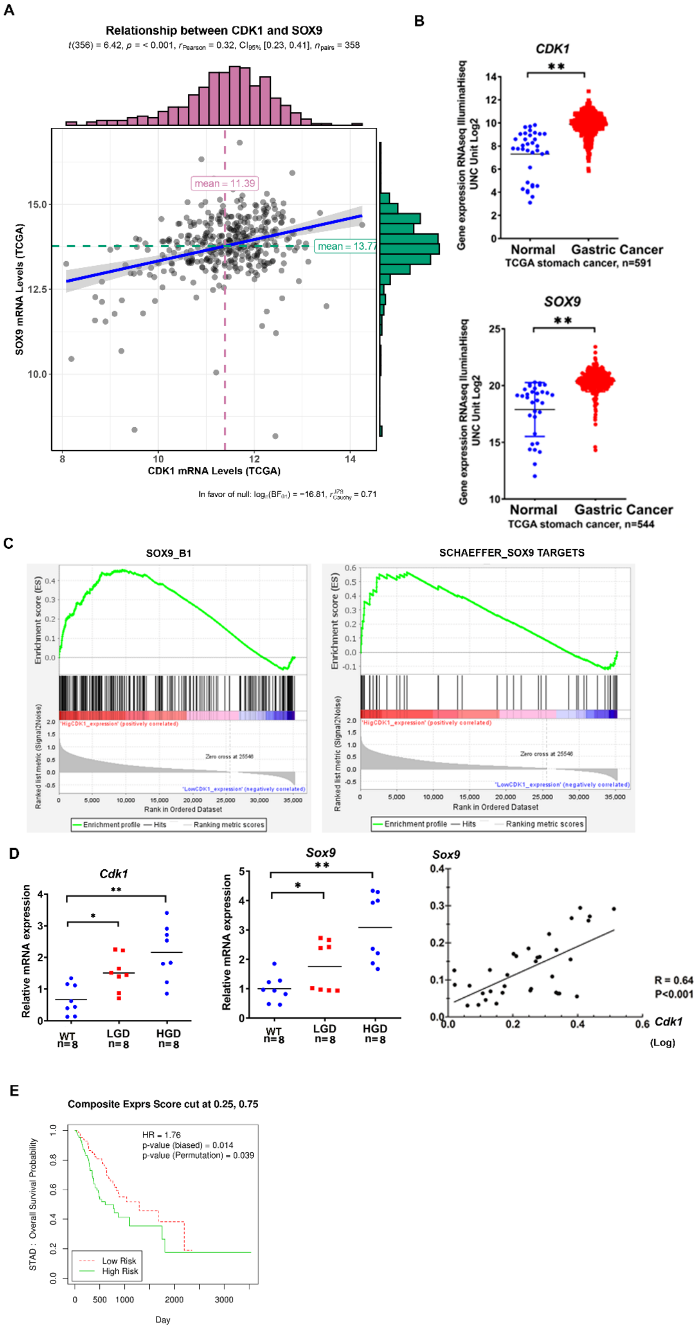

Supp. Figure 1: CDK1 and SOX9 correlation in gastric cancer. A positive correlation of *CDK1* and *SOX9* mRNA levels from TCGA dataset **(A).** Gene expression of *CDK1* and *SOX9* from RNAseq data from normal and tumor samples **(B)**. Gene set enrichment analysis (GSEA) for high and low *CDK1* expression in GC from TCGA dataset in SOX9 signatures, SOX9_B1 (NES: 1.56, FDR q value: 0.01) and Schaeffer SOX9 targets (NES: 1.67, FDR q value: 0.09) **(C).** mRNA transcripts of *Cdk1* and *Sox9* in mouse gastric tissue samples (antrum), control (n=8), low-grade dysplasia (LGD) (n=8), and high-grade dysplasia (HGD) (n=8), and the correlation of *Cdk1* and *Sox9* expression levels in mouse gastric samples **(D).** Kaplan-Meier survival curve analysis of CDK1 and SOX9 co- expression in STAD dataset **(E).** (*p<0.05, **p<0.01, ***p<0.001)

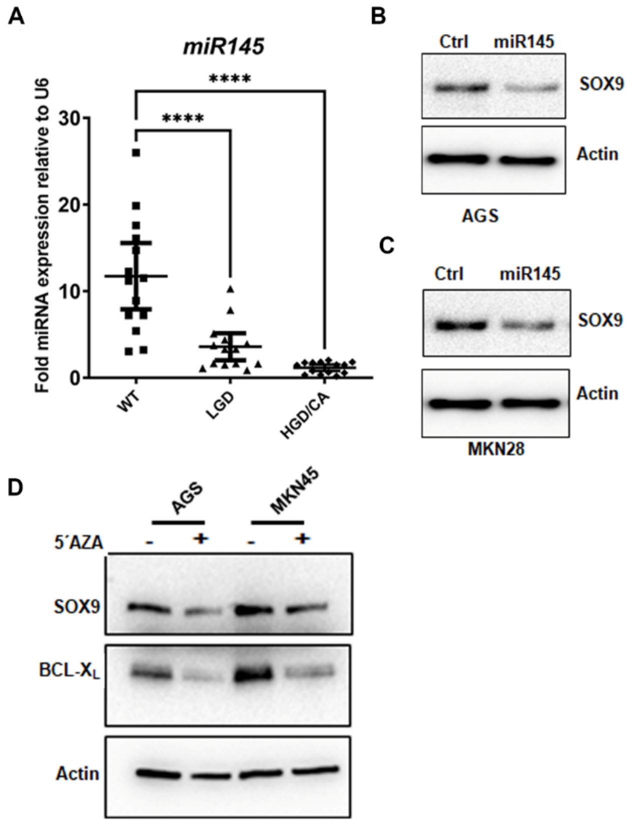

Supp. Figure 2: miR-145 is downregulated in GC. mRNA transcripts of *miR-145* in mouse gastric samples, WT (n=14), low-grade dysplasia (LGD) (n=14), and high-grade dysplasia (HGD/CA) (n=14) **(A);** ****p<0.0001. AGS and MKN28 cells were transfected with miR-145 mimic for 72h then SOX9 protein levels were detected by western blot analysis. A representative β-actin is shown as an internal control in WB **(B, C)**. AGS and MKN45 cells were treated with 5' AZA for 72h then SOX9 and BCL-xL protein levels were detected by western blot analysis. A representative β-actin is shown as an internal control in WB **(D).**

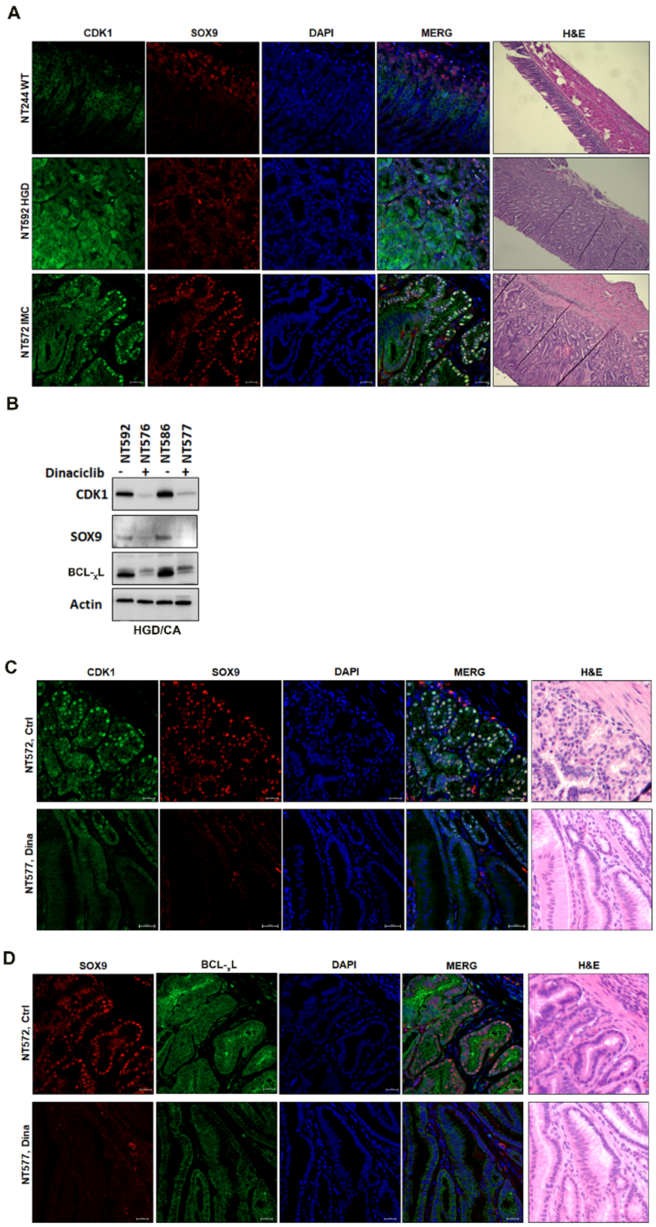

Supp. Figure 3: CDK1 inhibition attenuated *Bcl2l1* (Bcl-xL) mRNA expression and protein levels in the *Tff1* knockout mouse model. Immunofluorescence staining of CDK1and SOX9 in wild-type controls (NT244 WT), *Tff1* knockout mice with high-grade dysplasia (NT592 HGD), and intramucosal carcinoma (NT572 IMC), scale bar = 20µm. Representative H&E-stained images are included to show morphology, scale bar= 50 µm. DAPI is used for nuclear staining **(A)**. *Tff1* knockout mice (NT592, NT576, NT586, NT577) control or treated with Dinaciclib (Dina) for 4 weeks and whole cell lysate was collected from the tissues and subjected to western blot analysis, membranes were probed with CDK1, SOX9 and BCL-xL, and β-actin **(B)**. Immunofluorescence staining of CDK1, SOX9 and BCL-xL, scale bar = 20µm. DAPI is used for nuclear staining. The identical slide sections were used for both staining procedures **(C, D)**. NT572 was utilized in panels A, C, and D, while NT577 was used in panels C and D. The H&E-stained images were acquired from the same slide.

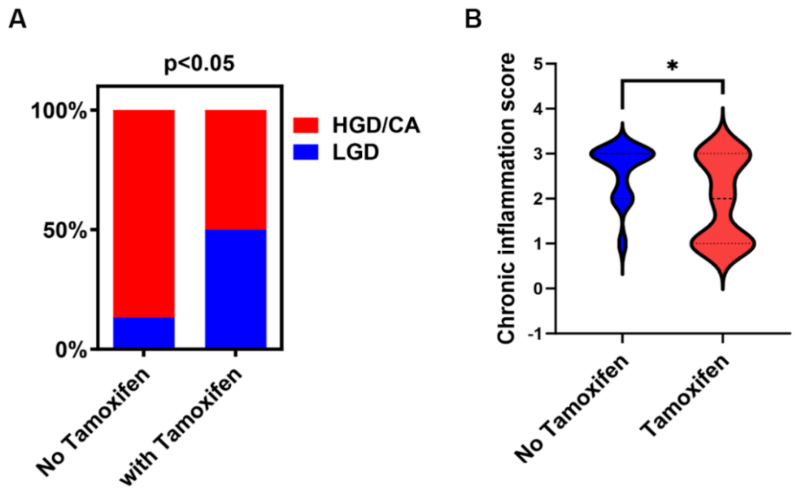

Supp. Figure 4: *Krt19^{CreERT}/Cdk1$^{flox/flox}$ / Tff1$^{-/-}$* mouse model attenuated tumor progression and increased the chronic inflammation. Mice were randomized and received either vehicle or tamoxifen (50 mg/kg/IP) daily for 10 days, at the age of 8-10 weeks. The mice were euthanized at 6-7 months of age, and tissues were collected for histological evaluation **(A)**. Chronic inflammation scores were evaluated based on inflammatory cell infiltration and mucosal changes **(B)**. No tamoxifen (LGD= 2, HGD/CA= 13), Tamoxifen (LGD= 7, HGD/CA= 7). *P<0.05. LGD, low-grade dysplasia; HGD, high-grade dysplasia; CA, carcinoma

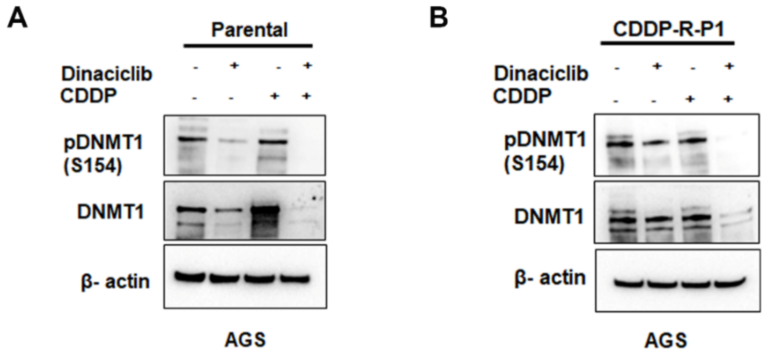

Supp. Figure 5: Dinaciclib sensitizes AGS cells to CDDP. AGS parental cells and CDDP-R cells (P1) were treated with cisplatin 10 uM and Dinaciclib 20nM, WCL was collected and subjected to western blots. Membranes were probed with pDNMT1 (S154), DNMT1 and β-actin **(A, B)**.
