# Supplementary material for: CDK1 drives SOX9-mediated chemotherapeutic resistance in gastric cancer
Source: J Exp Clin Cancer Res. 2025 Oct 8;44:284. doi: 10.1186/s13046-025-03523-3 (PMC12506374; doi:10.1186/s13046-025-03523-3)
Supplement: Supplementary file 2 — Supplementary Material 2 [file 13046_2025_3523_MOESM2_ESM.pdf]

Supplementary Table 1. The expression of top 10 SOX9 target genes in STAD

Gene	Normal Sample Avg	SD	Cancer Sample Avg	SD	FC	log2 Fold Change	p value	Critical Value	Benjamini-Hochberg Adjusted P value	Significant using an FDR of 0.05?
HOXB9	2.146229	4.696659	19.38592	28.46287	8.032553	3.175134	4.21E-23	0.005	1.60E-54	Yes
HOXA10	0.762486	1.395858	13.78347	13.75674	17.07702	4.176085	1.6E-55	0.01	2.11E-22	Yes
SLC39A8	9.431914	6.928443	24.91158	23.67124	1.641201	1.401194	3.99E-16	0.015	3.36E-17	Yes
BCL2L1	60.97494	31.61072	134.697	71.76857	1.209054	1.143429	1.01E-17	0.02	9.99E-16	Yes
FGFR3	23.325	36.0126	30.34464	39.90579	0.300949	0.379565	0.27872	0.025	6.98E-12	Yes
EIF3A	77.39957	27.17824	121.4365	40.34754	0.568956	0.649805	1.47E-11	0.03	2.45E-11	Yes
HMGN3	69.16626	28.76581	101.9253	74.89891	0.473627	0.559371	7.15E-07	0.035	1.02E-06	Yes
DUSP6	29.75449	23.76111	40.73498	29.79278	0.369037	0.453161	0.013717	0.04	0.017146786	Yes
TRIM2	35.27143	11.84387	56.95044	40.00249	0.614634	0.691207	3.49E-12	0.045	0.069217469	No
TLE1	32.77591	20.13637	39.52024	17.68936	0.205771	0.269956	0.062296	0.05	0.278720071	No

Supplementary Table 2. Dinaciclib treatment in conditional TFF1 knockout mice

ID	Diagnosis	Treatment	Gender	Genotype	Age (months)
NT 584 ST	Normal Stomach	Ctrl	FEMALE	*Tff1* $^{-/-}$	8.4
NT 586 ST	Intramucosal Adenocarcinoma	Ctrl	FEMALE	*Tff1* $^{-/-}$	8.4
NT 592 ST	High Grade Dysplasia	Ctrl	FEMALE	*Tff1* $^{-/-}$	8.4
NT 572	Intramucosal Adenocarcinoma	Ctrl	FEMALE	*Tff1* $^{-/-}$	11.4
NT 576 ST	Focal High-Grade Dysplasia	Dinaciclib	MALE	*Tff1* $^{-/-}$	10.2
NT 577 ST	Adenocarcinoma	Dinaciclib	MALE	*Tff1* $^{-/-}$	10.2
NT 578 ST	Antral hyperplasia and chronic gastritis	Dinaciclib	MALE	*Tff1* $^{-/-}$	10.2
NT 579	Intramucosal Adenocarcinoma	Dinaciclib	FEMALE	*Tff1* $^{-/-}$	10.2
NT 580 ST	Low Grade Dysplasia	Dinaciclib	FEMALE	*Tff1* $^{-/-}$	10.2
NT 581 ST	Normal Stomach	Dinaciclib	FEMALE	*Tff1* $^{-/-}$	10.2
NT 582 ST	Normal Stomach	Dinaciclib	FEMALE	*Tff1* $^{-/-}$	10.2
NT 583 ST	High Grade Dysplasia	Dinaciclib	FEMALE	*Tff1* $^{-/-}$	10.2

Supplementary Table 3. Conditional *Cdk1*/*Tff1* knockout mice

ID	Diagnosis	Treatment	Gender	Genotype	Age (months)
CKF167	Intramucsoal Carcinoma	vehicle	M	cre+/+, cdk1 flox +/+ tff1 -/-	6.54
CKF168	Invasive Adenocarcinoma	vehicle	M	cre+/+, cdk1 flox +/+ tff1 -/-	6.54
CKF166	HGD	vehicle	M	cre+/+, cdk1 flox +/+ tff1 -/-	6.54
CKF169	HGD	vehicle	F	cre+/+, cdk1 flox +/+ tff1 -/-	5.42
CKF170	LGD	vehicle	F	cre+/+, cdk1 flox +/+ tff1 -/-	5.42
CKF164	LGD	vehicle	F	cre+/+, cdk1 flox +/+ tff1 -/-	6.54
CKF172	LGD	Tamoxifen 50mg/kg for 10 days	M	cre+/+, cdk1 flox +/+ tff1 -/-	6.38
CKF173	LGD	Tamoxifen 50mg/kg for 10 days	M	cre+/+, cdk1 flox +/+ tff1 -/-	6.38
CKF175	Invasive Adenocarcinoma	Tamoxifen 50mg/kg for 10 days	M	cre+/+, cdk1 flox +/+ tff1 -/-	6.38
CKF176	LGD	Tamoxifen 50mg/kg for 10 days	M	cre+/+, cdk1 flox +/+ tff1 -/-	6.38
CKF171	LGD	Tamoxifen 50mg/kg for 10 days	F	cre+/+, cdk1 flox +/+ tff1 -/-	6.38
CKF249	Invasive Adenocarcinoma	vehicle	M	cre+/+, cdk1 flox +/+ tff1 -/-	7.13
CKF250	Intramucosal Adenocarcinoma	vehicle	M	cre+/+, cdk1 flox +/+ tff1 -/-	7.1
CKF251	Invasive Adenocarcinoma	vehicle	M	cre+/+, cdk1 flox +/+ tff1 -/-	7.1
CKF252	Invasive Adenocarcinoma	vehicle	M	cre+/+, cdk1 flox +/+ tff1 -/-	7.13
CKF253	Invasive Adenocarcinoma	vehicle	M	cre+/+, cdk1 flox +/+ tff1 -/-	7.13
CKF259	Invasive Adenocarcinoma	vehicle	F	cre+/+, cdk1 flox +/+ tff1 -/-	7.36
CKF260	Invasive Adenocarcinoma	vehicle	F	cre+/+, cdk1 flox +/+ tff1 -/-	7.36
CKF261	Intramucosal Adenocarcinoma	vehicle	F	cre+/+, cdk1 flox +/+ tff1 -/-	7.33
CKF262	Intramucosal Adenocarcinoma	vehicle	F	cre+/+, cdk1 flox +/+ tff1 -/-	7.36
CKF254	HGD	Tamoxifen 50mg/kg for 10 days	F	cre+/+, cdk1 flox +/+ tff1 -/-	7.13
CKF255	LGD	Tamoxifen 50mg/kg for 10 days	F	cre+/+, cdk1 flox +/+ tff1 -/-	7.13
CKF256	HGD	Tamoxifen 50mg/kg for 10 days	F	cre+/+, cdk1 flox +/+ tff1 -/-	7.13
CKF257	HGD	Tamoxifen 50mg/kg for 10 days	F	cre+/+, cdk1 flox +/+ tff1 -/-	7.13
CKF258	LGD	Tamoxifen 50mg/kg for 10 days	F	cre+/+, cdk1 flox +/+ tff1 -/-	7.1
CKF263	LGD	Tamoxifen 50mg/kg for 10 days	M	cre+/+, cdk1 flox +/+ tff1 -/-	7.36
CKF264	Invasive Adenocarcinoma	Tamoxifen 50mg/kg for 10 days	M	cre+/+, cdk1 flox +/+ tff1 -/-	7.36
CKF265	Intramucosal Adenocarcinoma	Tamoxifen 50mg/kg for 10 days	M	cre+/+, cdk1 flox +/+ tff1 -/-	7.33
CKF266	Invasive Adenocarcinoma	Tamoxifen 50mg/kg for 10 days	M	cre+/+, cdk1 flox +/+ tff1 -/-	7.33
CKF243	Invasive Adenocarcinoma	Tamoxifen 50mg/kg for 10 days	M	cre+/+, cdk1 flox +/+ tff1 -/-	7.69
CKF244	LGD	Tamoxifen 50mg/kg for 10 days	M	cre+/+, cdk1 flox +/+ tff1 -/-	7.69
CKF246	HGD	Tamoxifen 50mg/kg for 10 days	F	cre+/+, cdk1 flox +/+ tff1 -/-	7.73
CKF248	Invasive Adenocarcinoma	Tamoxifen 50mg/kg for 10 days	F	cre+/+, cdk1 flox +/+ tff1 -/-	7.73

Supplementary Table 4. PDXs information

	MOLECULAR/ ISTOLOGICAL ANALYSIS ON PDX				ORIGINAL TUMOR FEATURES					
CODE	HER2/ EGFR/ MET/ KRAS CNG (qReal Time PCR)	MSI STATUS	EBV	ISTOLOGY	TUMOR SITE	DIFFERENTIATION GRADE	pT	pN	pM	STAGE
**PDX498**	NO CNG	MSI	NEG/LOW	intestinal	CARDIAS	G4	T3	N3a	M1	IIIB
**PDX539**	EGFR 5-7 COPIES	MSS	NEG/LOW	diffuse	FUNDUS	ND	T3	N1	M0	IIB

Supplementary Table 5. Sequences for primers

Gene Name	Sequences		Assay
	Forward	Reverse	
mCdk1	TGGCCAGTGACTCTGTGTCT	TCCGTCGTAACCTGTTGAGT	qRT-PCR
mHprt	TAT GCC GAG GAT TTG GAA AA	ACA GAG GGC CAC AAT GTG AT	qRT-PCR
mBcl2l1	CTCTTCAGGGGAAACTGAGG	TGCTCACTTACTGGGTCTGC	qRT-PCR
mSox9	TCCACGAAGGGTCTCTTCTC	AGGAAGCTGGCAGACCAGTA	qRT-PCR
CDK1	CAACTCCATAGGTACCTTCTCCA	GCGGAATAATAAGCCGGGAT	qRT-PCR
BCL2L1	GAGCTGGTGGTTGACTTTCTC	TCCATCTCCGATTCAGTCCCT	qRT-PCR
HPRT1	TTG GAA AGG GTG TTT ATT CCT CA	TCC AGC AGG TCA GCA AAG AA	qRT-PCR
SOX9	GTAATCCGGGTGGTCCTTCT	GACGCTGGGCAAGCTCT	qRT-PCR
U6	GCAAGGATGACACGCAAAT		qRT-PCR
MiR145	GTCCAGTTTTCCCAGGAATCCCT		qRT-PCR
BCL2L1-P1	TGCAGATCTGAGGCAGTTTCC	AGGGACTTCTCAATGGGGTTC	ChIP
BCL2L1-P2	GTTCTGGGCCTTTGGGGAAT	TGTGATGTTGAAGGCCGGAG	ChIP
BCL2L1-P3	GAAGCTACCGGGCCGATG	GGACGGCGAAGGCTCCTATT	ChIP

Supplementary Table 6. Source and dilutions of antibodies

Antibody	Source	Catalogue Number	Application	Dilution
Rabbit CDK1	Cell Signaling	#28439	WB	(1:1000)
Mouse CDK1	Cell Signaling	#9116	WB	(1:400)
Rabbit CDK1	AbCam	ab133327	IHC	(1:100)
Rabbit CDC2/CDK1	Novus	#NBP1-85729	IF	(1:100)
Mouse SOX9	Novus	#NBP2-52943	WB, ChIP	(1:1000), (1:100)
Rabbit pDNMT1 (S154)	Invitrogen	# PA5-12963	WB	(1:1000)
Rabbit DNMT1	Cell Signaling	#5032	WB	(1:100)
β-actin	Sigma-Aldrich	A1978	WB	(1:4000)
HRP-coupled anti-mouse,anti-rabbit	Promega	W4021, W4011	WB	(1:4000)
Rabbit BCLXL	Cell Signaling	#2764S	WB, IF	(1:2000), (1:300)
Rabbit PARP	Cell Signaling	#9542S	WB	(1:1000)
Rabbit Cl-PARP	Cell Signaling	# 5625S	WB	(1:300)
Mouse Ki67, eBioscience	ThermoFisher	# 14-5699-82	IF	(1:500)
Rabbit Cl-Caspase3	Cell Signaling	# 9664S	IF	(1:300)
Normal IgG	EMD Millipore corp.	#3281600	ChIP	/
Goat anti-Rabbit IgG (H+L) Highly Cross-Adsorbed Secondary Antibody, Alexa Fluor™ 488		# A-11011	IF	(1:4000)
Goat anti-Rabbit IgG (H+L) Cross-Adsorbed Secondary Antibody, Alexa Fluor™ 568	ThermoFisher	# A-11034	IF	(1:4000)

Supplementary Table 7. Reagents and Kits

Name	Source	Catalogue Number
EZ DNA Methylation-Gold Kit	ZYMO RESEARCH	D5005
Zymo-Spin ChIP Kit	ZYMO RESEARCH	D5210
QuikChange II XL Site-Directed Mutagenesis Kit	Agilent	200521
Platinum PCR SuperMix High Fidelity	ThermoFisher	12532016
PyroMark Gold Q96 Reagents	Qiagen	972804
Dinaciclib	Selleckchem	S2768
Cisplatin	MedChemExpress	HY-17394
5-Azacytidine	MedChemExpress	HY-10586
Tamoxifen	Sigma	T5648
Cdc2-HA	Addgene	#1888
SOX9-3'UTR	Established in lab	
pCMV-Tag2-Sox9 T236A	Addgene	#111455
pCMV-Tag2-Sox9	Established in lab	
ON-TARGETplus Non-targeting Control siRNAs	Horizon	D-001810-02-05
ON-TARGETplus Human CDK1 (983) siRNA - SMARTpool, 5 nmol	Horizon	J-003224-13-0005
ON-TARGETplus Human SOX9 (6662) siRNA - Individual, 5 nmol	Horizon	J-021507-05-0005
siDNMT1	Santa Cruz	sc-35204
pRP[Pro]-hRluc-{BCL2L 1 _FL}>Luciferase	vectorbuilder	VB230720-1456fsu
pRP[Pro]-hRluc/Pu ro-{BCL2L 1 _R3}>Luciferase	vectorbuilder	VB230720-1454vnn
DharmaFECT 1 Transfection Reagent	Horizon	T-2001-02
FuGene 4K Transfection reagent	FuGene	4K-1000
